# The independent value and clinical significance of angio-based microvascular resistance in predicting adverse cardiovascular events in patients with acute ST-elevation myocardial infarction

**DOI:** 10.3389/fcvm.2025.1637251

**Published:** 2025-09-25

**Authors:** Zichen Han, Shiyi Gao, Yiliang Yan, Xuemin Hu, Chong Wang, Zengwei Cheng, Sigan Hu

**Affiliations:** ^1^ Bengbu Medical University Graduate School, Bengbu, Anhui, China; ^2^ Department of Cardiology, Suzhou First People’s Hospital, Suzhou, Anhui, China; ^3^ Department of Cardiology, The First Affiliated Hospital of Bengbu Medical University, Bengbu, Anhui, China; ^4^ Department of Neurology, Suzhou First People’s Hospital, Suzhou, Anhui, China; ^5^ Department of Cardiology, Wuhe First People’s Hospital, Bengbu, Anhui, China

**Keywords:** ST-segment elevation myocardial infarction, coronary microvascular dysfunction, angio-based microvascular resistance, quantitative flow ratio, percutaneous coronary intervention, major adverse cardiovascular events

## Abstract

**Background:**

Angio-based microvascular resistance (AMR) may influence the incidence of major adverse cardiovascular events (MACE) in patients with ST-segment elevation myocardial infarction (STEMI) after percutaneous coronary intervention (PCI). However, its value as an independent predictive marker remains unclear.

**Methods:**

This study included 483 patients diagnosed with STEMI who underwent PCI between January 2021 and July 2023. The patients were classified into high and low AMR groups based on the AMR threshold. The relationship between AMR and MACE was assessed using multivariate logistic regression analysis, and the cumulative incidence of MACE was analyzed using Kaplan–Meier survival curves. Additionally, receiver operating characteristic (ROC) curves were used to determine the optimal cutoff value for AMR and its predictive efficacy.

**Results:**

During the 12-month follow-up period, the cumulative incidence of MACE was significantly higher in the high AMR group than in the low AMR group (*P* < 0.0001). Multivariate logistic regression analysis indicated that AMR was an independent predictor of MACE (HR = 1.085, 95% CI: 1.037–1.248, *P* < 0.001). Kaplan–Meier survival curve analysis further validated a poorer prognosis in the high AMR group, with a significantly increased risk of MACE. ROC curve analysis established the optimal cutoff value of AMR at 246.5 mmHg·s/m, at which the sensitivity for predicting MACE was 0.98, with a specificity of 0.67 and an area under the curve of 0.889, indicating good predictive performance. Additionally, diabetes, hyperlipidemia, and elevated levels of N-terminal pro B-type natriuretic peptide (NT-proBNP) were significantly associated with the occurrence of MACE.

**Conclusion:**

AMR holds independent prognostic value for predicting MACE, with an optimal cutoff of 246.5 mmHg·s/m, facilitating early risk stratification by identifying high-risk patients. Additionally, diabetes, hyperlipidemia, and elevated NT-proBNP levels were significantly associated with an increased risk of MACE. A low postoperative quantitative flow ratio also correlated with a higher MACE risk, further highlighting the impact of coronary blood flow restoration on patient outcomes.

## Background

Coronary artery disease (CAD) significantly contributes to global mortality rates (1). Percutaneous coronary intervention (PCI) is a well-established and extensively utilized treatment for CAD in clinical practice (2). Nevertheless, even with successful PCI, the incidence of complications remains considerable and significantly affects patient outcomes (3–6). Emerging research suggests that adverse events following PCI are influenced not only by the extent of epicardial coronary stenosis and the effectiveness of revascularization but also by coronary microvascular dysfunction (CMD), which has emerged as a crucial element in the management of CAD (6–8).

The coronary microcirculation, consisting of arterioles, capillaries, and venules (with diameters <500 µm), functions as the terminal segment of the cardiac blood supply system, playing an essential role in maintaining myocardial perfusion and function (6, 9, 10). Studies have shown that despite the successful reopening of major coronary arteries following PCI, persistent myocardial ischemia and reperfusion injury may still develop. This phenomenon may be attributed to underlying CMD (11).

Currently, the evaluation of coronary microcirculation function primarily involves two categories of diagnostic methods: invasive and non-invasive techniques (12–14). Non-invasive diagnostic techniques, such as cardiac magnetic resonance imaging (CMR), can be employed to evaluate the microcirculatory status in patients (12). However, its applicability to certain patient populations is somewhat limited due to the high cost of the equipment and the requirement for patients to perform multiple breath-holds during the procedure (15, 16). Invasive procedures, such as IMR, inherently involve certain risks, and the administration of adenosine during the procedure may potentially induce arrhythmia (17). In recent years, the index Angio-based microvascular resistance (AMR), which serves as a novel indicator for assessing coronary microcirculatory resistance, has garnered increasing attention in clinical research. It is derived from standard coronary angiographic images and does not necessitate the use of additional guidewires or pharmacological agents. Therefore, it offers a higher level of safety. The quantitative flow ratio (QFR) index associated with AMR can assess the hemodynamic significance of coronary artery stenosis, as confirmed by multiple studies that have directly compared it with fractional flow reserve (FFR).

At present, few people have studied the prognosis of patients from the perspectives of AMR and QFR. This study offers a comprehensive analysis of QFR and AMR in patients with ST-segment elevation myocardial infarction (STEMI), aiming to clarify the relationships between specific hemodynamic parameters, coronary lesion characteristics, and major adverse cardiovascular events (MACE). By identifying potential high-risk factors among patients with CAD, our objective was to develop more precise risk assessment tools and to facilitate the implementation of individualized therapeutic strategies in clinical practice.

## Methods

### Study design

The study recruited patients presenting with STEMI to the emergency departments of The First Affiliated Hospital of Bengbu Medical University and Suzhou First People's Hospital between January 1, 2021, and July 1, 2023, who underwent PCI. The inclusion criteria were as follows: (1) age 18 years or older, (2) a definitive diagnosis of STEMI, (3) successful PCI performed during the acute phase. Successful PCI was defined by a significant reduction in coronary stenosis and the attainment of thrombolysis in myocardial infarction (TIMI) grade 3 flow, indicating complete reperfusion, as confirmed by angiography. (4) Patients with single-vessel infarct-related STEMI only. The exclusion criteria comprised: (1) patients with a diagnosis of STEMI who declined further treatment or were unable to maintain follow-up due to individual circumstances; (2) CAG images that did not meet the required analytical standards, such as being blurry, having irregular data formats, incomplete imaging, significant vascular overlap or artifacts, or being single-view post-PCI; (3) patients with STEMI involving multiple infarct-related arteries; (4) patients who had experienced recent major bleeding events or had a predisposition to bleeding. The study protocol was approved by the ethics committees of The First Affiliated Hospital of Bengbu Medical University (Approval No.: KY046) and Suzhou First People's Hospital (Approval No.: SZYYLLky2024016), and all participants provided informed consent. This study complied with the Declaration of Helsinki and pertinent medical ethical guidelines, guaranteeing the voluntary participation of patients.

### Calculation of QFR and AMR

After reperfusion of the culprit vessel, single-view QFR and AMR analyses, guided by Murray's law, were performed using the QFR software (AngioPlus Gallery, Pulse Medical Technology Inc., Shanghai, China). The process is fully automated, allowing the software to accurately identify optimal arterial lumen contours. Manual adjustments were made only in cases where the automatic identification was suboptimal, ensuring minimal vessel overlap and optimal image clarity. The hyperemic flow velocity was calculated by dividing the vessel centerline length by the time required for the contrast agent to completely fill the vessel. The analytical framework employs comprehensive contrast enhancement and full-lumen exposure techniques to automatically delineate the boundaries of the vessel and its major branches. As previously described, the pressure gradient in bifurcated vessels was evaluated using Murray's bifurcation fractal law. The AMR was determined by the ratio of distal coronary pressure to hyperemic flow rate (18, 19). The calculation procedure and schematic diagram of AMR are detailed in Supplementary Material 1.

### Outcomes and follow-up

The main outcome measure was established as a combination of cardiac death or rehospitalization for heart failure within one year after undergoing PCI. Secondary endpoints encompassed the components of the primary endpoint and patient-centered adverse events, including all-cause mortality, recurrent myocardial infarction, and revascularization procedures. All clinical outcomes were evaluated according to standardized definitions established by the Academic Research Consortium. Cardiac death encompassed fatalities due to cardiac causes, unknown etiology, or indeterminate origin. Heart failure readmission was defined as a recent exacerbation of symptoms or a significant decline in cardiac function, characterized by a left ventricular ejection fraction of less than 50%, elevated B-type natriuretic peptide levels, or a diagnosis of heart failure at discharge (20). All incidents were independently evaluated by seasoned cardiologists in a blinded manner, and any disagreements were resolved through consensus among the experts. Follow-up data were collected through telephone interviews, outpatient records, and hospitalization documents.

### Statistical analysis

All statistical analyses were performed using SPSS 27.0 (IBM Corporation, Armonk, NY) and R 4.4.1 (The R Foundation for Statistical Computing). Continuous variables are reported as mean ± standard deviation (SD) or median (interquartile range, IQR), with normal distribution evaluated using the ShapiroWilk test. For variables that follow a normal distribution, the independent samples *t*-test was employed for between-group comparisons. In contrast, the Mann–Whitney *U* test was utilized for variables that do not follow a normal distribution. Categorical variables are reported as frequencies and proportions, and comparisons were conducted using either the chi-square test or Fisher's exact test. The independent association between AMR and MACE was assessed using a multivariate logistic regression model, with results presented as odds ratios (ORs) and 95% confidence intervals (CIs). All variables in the model underwent multicollinearity testing using the Variance Inflation Factor (VIF), confirming that multicollinearity levels were maintained within an acceptable range (VIF < 10). To ensure statistical robustness, we performed two-sided tests at a significance level of *P* < 0.05. The Kaplan–Meier method was used to assess the cumulative MACE incidence across varying AMR levels, with intergroup differences evaluated using the log-rank test. Receiver operating characteristic (ROC) curve analysis was performed to determine the optimal antimicrobial resistance (AMR) cutoff, and its discriminatory performance was assessed using the area under the curve (AUC), sensitivity, and specificity. The threshold for statistical significance was established at *P* < 0.05.

## Results

### Patient population

**Figure 1:**
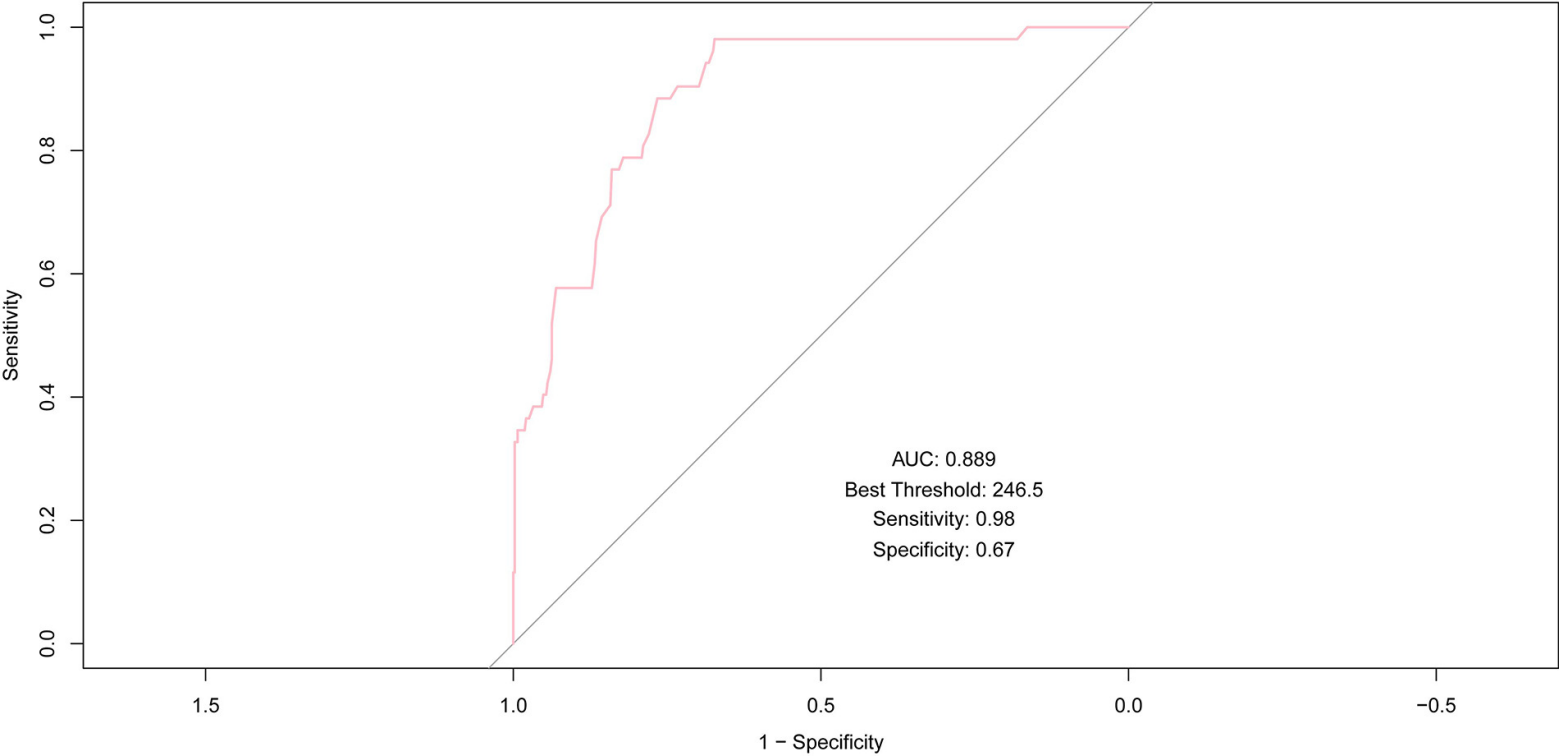
Flowchart for patient selection. MACE, major adverse cardiac events; QFR, quantitative flow ratio; PCI, percutaneous coronary intervention.

Between January 1, 2021, and July 1, 2023, 748 patients undergoing PCI were screened. Following stringent clinical evaluation, 192 patients who failed to meet the inclusion criteria were excluded, and an additional 73 patients were excluded due to suboptimal angiographic image quality. A total of 483 patients were incorporated into the final analyses. Patients were divided into two groups according to their QFR and AMR measurements: the non-MACE group (*n* = 431) and the MACE group (*n* = 52) (Figure 1).

### Baseline characteristics

There were significant differences in baseline characteristics between the non-MACE and MACE groups (Table 1). Patients with MACE were older (67.00 [54.75–75.50] vs. 64.00 [54.00–74.00], *P* = 0.025) and had a higher prevalence of hypertension (88.5% vs. 59.9%, *P* < 0.001), diabetes (63.5% vs. 33.0%, *P* < 0.001), and hyperlipidemia (84.6% vs. 64.5%, *P* = 0.004). N-terminal pro B-type natriuretic peptide (NT-proBNP) concentrations were markedly elevated in the MACE group compared to the non-MACE group (1,190.00 [355.50, 4,777.50] pg/ml vs. 243.10 [82.12, 911.33] pg/ml, *P* < 0.001). Additionally, monocyte and platelet counts were elevated in the MACE group (0.62 [0.45, 0.91] vs. 0.48 [0.37, 0.64], *P* = 0.003; 244.50 [215.25, 266.00] vs. 208.00 [167.50, 245.00], *P* < 0.001), while lymphocyte counts were reduced (1.36 [1.00, 1.77] vs. 1.89 [1.50, 2.55], *P* < 0.001). Aspirin use at discharge was significantly lower in the MACE group compared to the non-MACE group (94.2% vs. 99.1%, *P* = 0.006), while the numerical of value AMR was notably higher in the MACE group than in the non-MACE group (292.50 [265.00, 351.25] vs. 230.00 [199.00, 256.00], *P* < 0.001).

**Table 1:** Baseline characteristics of the study population.

Factors	Non-MACE Group (*N* = 431)	MACE Group (*N* = 52)	*P* value
Study population			
Age, years	64.00 [54.00, 74.00]	67.00 [54.75, 75.50]	0.025
Male, *n* (%)	318.0 (73.78%)	37 (71.15%)	0.369
Cardiovascular risk factors			
Hypertension	258.0 (59.86%)	46 (88.46%)	<0.001
Diabetes	142.0 (32.95%)	33 (63.46%)	<0.001
Hyperlipemia	278.0 (64.50%)	44 (84.62%)	<0.004
Stroke	73.0 (16.94%)	10 (19.23%)	0.679
Smoking	324.0 (75.17%)	44 (84.62%)	0.131
Previous stable angina pectoris	66.0 (15.31%)	9 (17.31%)	0.708
Previous PCI	26.0 (6.03%)	2 (3.85%)	0.524
Pain-to-balloon time	240.00 [137.50, 420.00]	376.00 [295.25, 591.00]	<0.001
Laboratory index			
cTnI, ng/L	0.91 [0.07, 9.29]	4.12 [0.19, 17.35]	0.084
NT-proBNP, pg/ml	243.10 [82.12, 911.33]	1,190.00 [355.50, 4,777.50]	<0.001
Creatinine, µmol/L	67.00 [55.00, 78.50]	77.50 [59.50, 97.50]	0.103
CK/CKMB	7.50 [5.36, 9.64]	7.33 [6.07, 11.21]	0.883
TC-C, mmol/L	4.64 [3.79, 5.50]	4.51 [3.87, 5.22]	0.896
TG, mmol/L	1.53 [1.04, 2.26]	1.36 [0.98, 1.95]	0.327
HDL-C, mmol/L	1.03 [0.88, 1.23]	1.04 [0.92, 1.24]	0.821
LDL-C, mmol/L	2.75 [2.19, 3.35]	2.67 [2.12, 3.35]	0.619
Inflammatory index			
Neutrophil, (10^9^)	6.32 [4.90, 7.62]	6.68 [5.45, 8.16]	0.103
Monocyte, (10^9^)	0.48 [0.37, 0.64]	0.62 [0.45, 0.91]	0.003
Platelet, (10^9^)	208.00 [167.50, 245.00]	244.50 [215.25, 266.00]	<0.001
Lymphocyte, (10^9^)	1.89 [1.50, 2.55]	1.36 [1.00, 1.77]	<0.001
Killip class			0.064
I	178 (41.30%)	17 (32.69%)	
II	32 (7.42%)	9 (17.31%)	
III	211 (48.96%)	24 (46.15%)	
IV	10 (2.32%)	2 (3.85%)	
Discharge medications			
Aspirin	427.0 (99.07%)	49 (94.23%)	0.006
Ticagrelor	290.0 (67.29%)	30 (57.69%)	0.167
Clopidogrel	141.0 (32.71%)	19 (36.54%)	0.580
Statins	425.0 (98.61%)	50 (96.15%)	0.190
ACEI/ARB	204.0 (47.33%)	24 (46.15%)	0.872
Beta-blocker	338.0 (78.42%)	42 (80.77%)	0.696
ARNi	97.0 (22.51%)	13 (25.00%)	0.685
SGLT2i	23.0 (5.34%)	2 (3.85%)	0.647
Spirolactone	167.0 (38.75%)	27 (51.92%)	0.067
Furosemide	149.0 (34.57%)	25 (48.08%)	0.055
Vascular-related characteristics of criminals			
Infarct-related artery			0.063
LAD	222 (51.51%)	31 (59.62%)	
LCX	58 (13.46%)	6 (11.54%)	
RCA	151 (35.03%)	15 (28.84%)	
Multivessel disease			0.144
1	102 (23.67%)	12 (23.08%)	
2	178 (41.30%)	23 (44.23%)	
3	151 (35.03%)	17 (32.69%)	
TIMI Flow Grade (initial)			0.184
0	357 (82.83%)	49 (94.23%)	
1	59 (13.69%)	1 (1.92%)	
2	7 (1.62%)	0 (0%)	
3	8 (1.86%)	2 (3.85%)	
TIMI Flow Grade (post)			0.053
0	0 (0%)	0 (0%)	
1	0 (0%)	0 (0%)	
2	0 (0%)	0 (0%)	
3	432 (100%)	52 (100%)	
QFR	0.93 [0.87, 0.97]	0.90 [0.77, 0.97]	0.174
△QFR	0.05 [0.02, 0.12]	0.10 [0.02, 0.22]	0.054
AMR	230.00 [199.00, 256.00]	292.50 [265.00, 351.25]	<0.001

Values are expressed as mean ± SD, median [IQR], or *n* (%).

MACE, major adverse cardiac events; PCI, percutaneous coronary intervention; cTnI, cardiac troponin I; NT-proBNP, N-terminal pro B-type natriuretic peptide; CK/CKMB, creatine kinase/creatine kinase-MB; TC-C, total cholesterol; TG, triglycerides; HDL-C, high-density lipoprotein cholesterol; LDL-C, low-density lipoprotein cholesterol; ACEI, angiotensin-converting enzyme inhibitor; ARB, angiotensin receptor blocker; ARNi, angiotensin receptor-neprilysin inhibitor; SGLT2i, sodium-glucose co-transporter 2 inhibitor; LAD, left anterior descending; LCX, left circumflex; RCA, right coronary artery; TIMI, thrombolysis in myocardial infarction; QFR, quantitative flow ratio; AMR, Angio-based microvascular resistance;.

### Multivariate logistic regression analysis of MACE events

The analysis of baseline characteristics uncovered statistically significant disparities in clinical parameters between the MACE and non-MACE groups. A multivariate logistic regression analysis was utilized to evaluate the independent predictive significance of these variables, while accounting for potential confounders.

The multivariate logistic regression analysis identified several variables as significant predictors of MACE (Table 2). Advanced age (OR: 1.310, 95% CI: 1.102–1.564; *P* = 0.033) and an elevated neutrophil count (OR: 1.807, 95% CI: 1.228–2.658; *P* = 0.003) were identified as significant risk factors.

**Table 2:** Multivariate logistic regression analysis of MACE events.

Characteristics	OR	95% CI	*P* value
Age	1.310	1.102–1.564	0.033
Neutrophil	1.807	1.228–2.658	0.003
Platelet	1.032	1.012–1.052	0.002
Lymphocyte	0.839	0.762–0.964	0.001
Killip class	1.072	1.028–1.153	0.027
NT-proBnP	1.231	1.149–1.437	<0.001
QFR	0.674	0.519–0.874	0.014
AMR	1.085	1.037–1.248	<0.001

QFR, quantitative flow ratio; AMR, Angio-based microvascular resistance; OR, odds ratio; CI, confidence interval; MACE, major adverse cardiac events; NT-proBnP N-terminal pro B-type natriuretic peptide.

An elevated platelet count (OR: 1.032, 95% CI: 1.012–1.052; *P* = 0.002) was significantly associated with an increased risk of MACE, whereas a higher lymphocyte count (OR: 0.839, 95% CI: 0.762–0.964; *P* = 0.001) demonstrated a protective effect. Furthermore, an elevated Killip class (OR: 1.072, 95% CI: 1.028–1.153; *P* = 0.027) and increased NT-proBNP levels (OR: 1.231, 95% CI: 1.149–1.437; *P* < 0.001) were independently associated with a higher incidence of MACE. In contrast, the high-value QFR demonstrated a significant association with a reduced risk of MACE (OR, 0.674; 95% CI, 0.519–0.874; *P* = 0.014). Additionally, an elevated AMR (OR: 1.085, 95% CI: 1.037–1.248; *P* < 0.001) was significantly correlated with an increased incidence of MACE. All the variables listed above demonstrated statistical significance, with *P*-values <0.05.

After conducting the multivariate logistic regression analysis, an ROC curve analysis was carried out to evaluate the model's ability to discriminate MACE, and the AUC was calculated.

**Figure 2:**
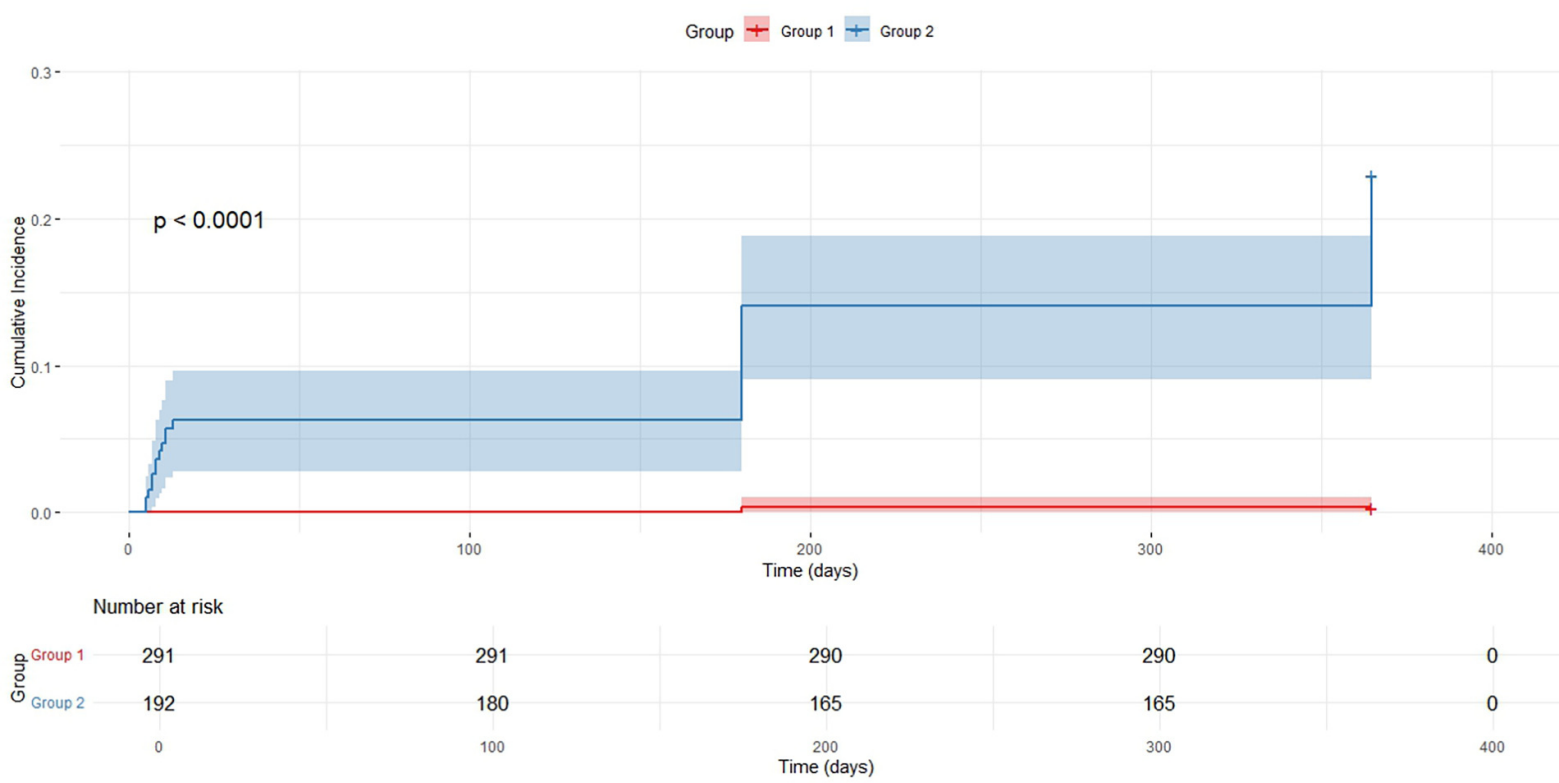
ROC curves for predictive variables of MACE; ROC, receiver operating characteristic; QFR, quantitative flow ratio; AMR, angio-based microvascular resistance; MACE, major adverse cardiac events; NT-proBNP, N-terminal pro B-type natriuretic peptide.

The ROC curve analysis of the eight variables revealed varying degrees of predictive accuracy for MACE (Figure 2). AMR exhibited the highest predictive accuracy (AUC = 0.889), followed by lymphocyte count (AUC = 0.762), indicating a strong discriminatory capacity. NT-proBNP (AUC = 0.697) and platelet count (AUC = 0.658) demonstrated moderate predictive accuracy for major adverse cardiac events (MACE). Conversely, QFR (AUC = 0.583), neutrophil count (AUC = 0.573), age (AUC = 0.529), and Killip classification (AUC = 0.48) exhibited limited predictive value, with AUC values approaching 0.5, suggesting nearly random classification.

AMR demonstrated robust predictive accuracy for MACE, achieving an AUC of 0.889, which underscores its strong discriminative power. At an optimal threshold of 246.5, AMR attained a sensitivity of 0.98 and a specificity of 0.67, further emphasizing its effectiveness in predicting outcomes, especially in identifying high-risk patients.

### Multivariate logistic regression analysis of AMR

**Figure 3:**
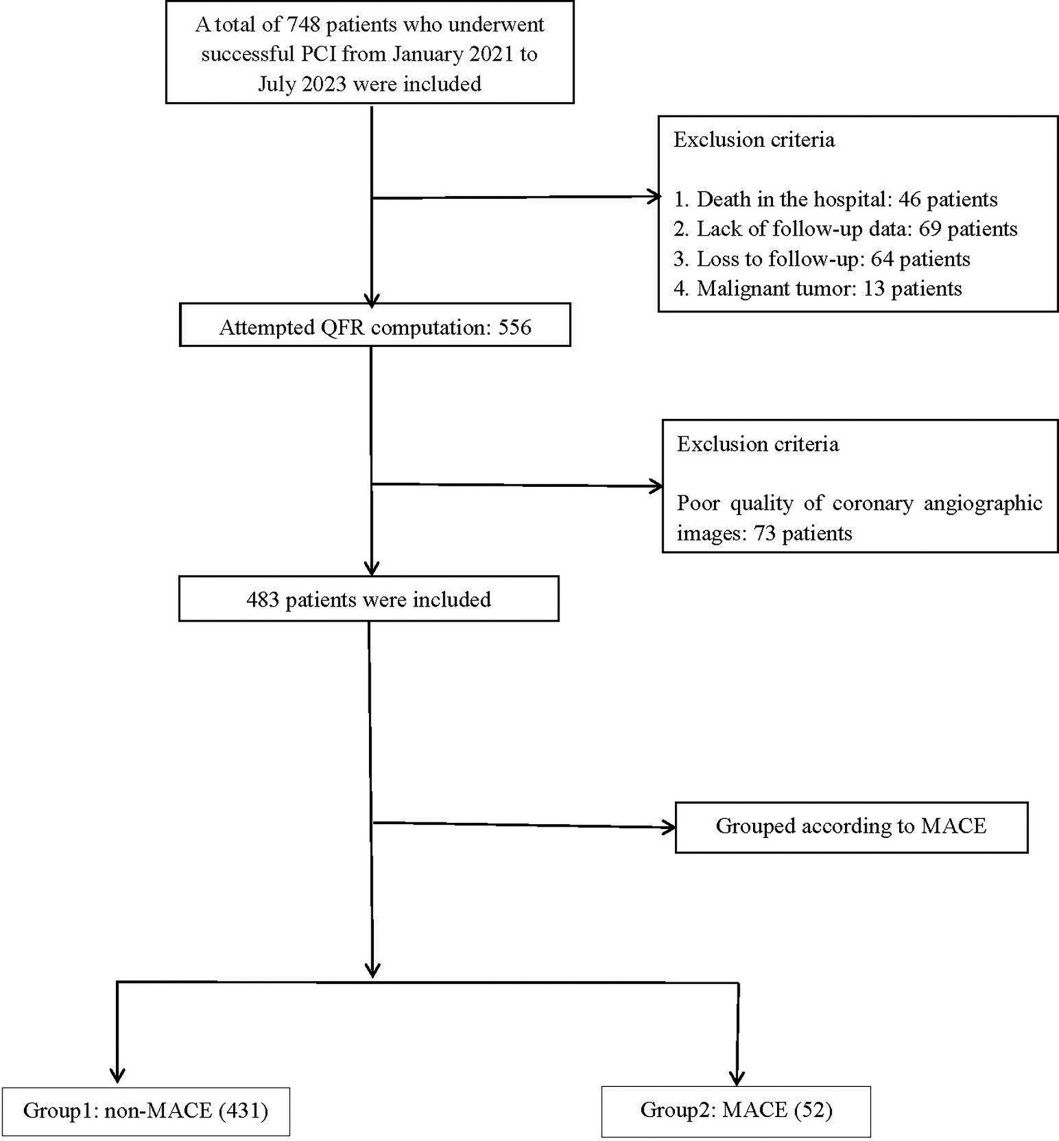
ROC curve of AMR for predicting MACE; AUC, area under the curve; ROC, receiver operating characteristic; MACE, major adverse cardiac events.

In multivariate analysis, AMR achieved the highest AUC (0.889), indicating its potential role in predicting MACE (Figure 3). To establish AMR as an independent predictor, it was integrated into a multivariate logistic regression model to assess its independent predictive value for MACE.

In the multivariate logistic regression analysis of AMR (Table 3), several factors were found to be significantly associated with an increased risk of AMR: age (OR: 1.597, 95% CI: 1.228–1.967, *P* = 0.002), diabetes (OR: 2.727, 95% CI: 2.321–5.132; *P* = 0.017), hyperlipidemia (OR: 2.119, 95% CI: 2.051–5.489; *P* < 0.001), NT-proBNP (OR: 1.002, 95% CI: 1–1.258; *P* = 0.014), longer reperfusion time (OR: 1.018, 95% CI: 1.003–1.034; *P* = 0.023), CK/CKMB ratio (OR: 1.356, 95% CI: 0.524–2.188; *P* = 0.001), platelet count (OR: 1.095, 95% CI: 1.015–1.175; *P* = 0.020), smoking history (OR: 15.337, 95% CI: 5.083–25.591; *P* = 0.003), and postoperative QFR (OR: 1.340, 95% CI: 1.267–1.769; *P* < 0.001). In contrast, a higher lymphocyte count (OR: 0.876, 95% CI: 0.659–0.983; *P* = 0.030) was identified as a significant protective factor against AMR.

**Table 3:** Multivariate logistic regression analysis of AMR.

Characteristics	OR	95% CI	*P* value
Age	1.597	1.228–1.967	0.002
Diabetes	2.727	2.321–5.132	0.017
Hyperlipemia	2.119	2.051–5.489	<0.001
NT-proBnP	1.002	1–1.258	0.014
Pain-to-balloon time	1.018	1.003–1.034	0.023
CK/CKMB	1.356	0.524–2.188	0.111
Platelet	1.095	1.015–1.175	0.020
Smoking	15.337	5.083–25.591	0.003
QFR	1.340	1.267–1.769	<0.001
Lymphocyte	0.876	0.659–0.983	0.030

QFR, quantitative flow ratio; AMR, Angio-based microvascular resistance; NT-proBNP, N-terminal pro B-type natriuretic peptide; OR, odds ratio; CI, confidence interval.

### Kaplan–Meier survival curve analysis

Participants were divided into two groups according to the optimal AMR cutoff value of 246.5 mmHg·s/m: the low AMR group (AMR < 246.5 mmHg·s/m, Group 1) and the high AMR group (AMR ≥ 246.5 mmHg·s/m, Group 2). A KaplanMeier survival analysis was conducted to assess the influence of AMR levels on the incidence of MACE and to compare the cumulative survival rates between the two groups.

**Figure 4:**
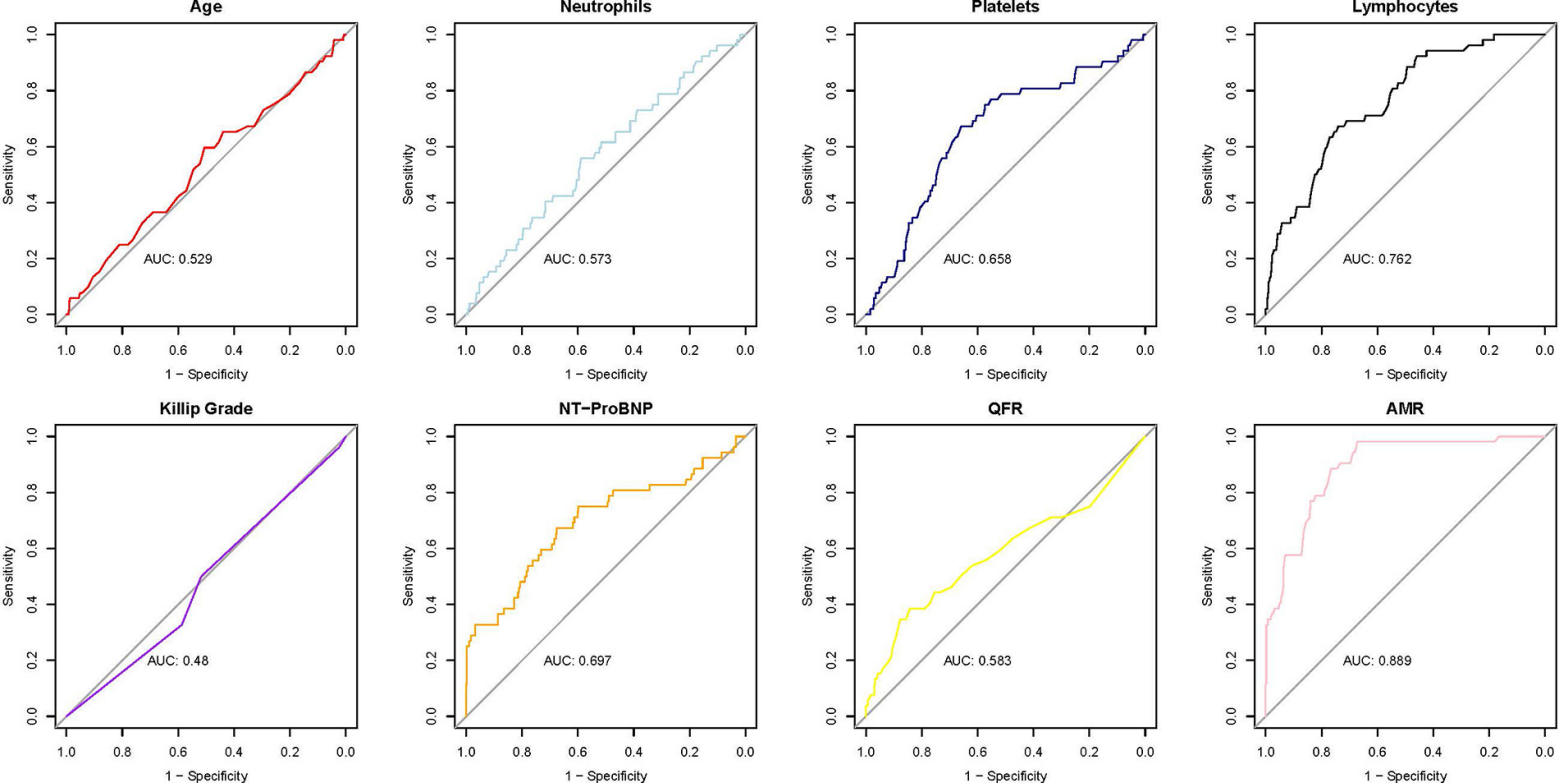
Kaplan–Meier survival curve comparison between two groups.

Kaplan–Meier survival curve analysis revealed a statistically significant difference in the cumulative incidence of MACE between patients with low and high AMR (*P* < 0.0001) (Figure 4). The cumulative incidence of MACE was consistently higher in the high AMR group than that in the low AMR group during the follow-up period, with a marked divergence in the survival curves observed after approximately 200 days. This finding suggests an association between elevated AMR and increased MACE risk.

## Discussion

This research validated AMR as a reliable and independent predictor of MACE in individuals with STEMI. The Kaplan–Meier survival analysis demonstrated a markedly the cumulative occurrence of MACE in patients with elevated AMR levels compared to those with lower levels, underscoring the potential value of AMR as a biomarker for cardiovascular risk stratification.

Our study findings established that an AMR threshold of ≥246.5 mmHg·s/m correlates with a significantly elevated risk of MACE, consistent with the findings of Luo et al. (21), who reported an increased risk of heart failure at AMR ≥250 mmHg·s/m. Both studies corroborate the link between elevated AMR levels and negative cardiovascular outcomes. However, the optimal AMR threshold may vary due to differences in study design, population characteristics, follow-up duration, and statistical methods. Further analysis indicated that, in addition to AMR, factors such as age, neutrophil count, platelet count, lymphocyte count, Killip classification, and NT-proBNP levels were significantly associated with MACE, which is partially consistent with the findings of Luo et al. highlighted the prognostic value of the postoperative QFR and coronary flow velocity (CFV), particularly when the QFR/AMR ratio was combined with CFV, demonstrating a stronger association with short-term heart failure. In contrast, the present study focused on the independent predictive value of AMR for MACE. Moreover, Qian et al. (22) indicated that AMR is a significant predictor of MACE, particularly all-cause mortality and heart failure readmissions. However, there is a discrepancy in the AMR threshold values: Qian et al. established a threshold of 255 mmHg·s/m, while this study identified an optimal threshold of 246.5 mmHg·s/m. These differences can likely be attributed to variations in sample characteristics and statistical methodologies. Specifically, the current study primarily included patients with multivessel disease, who had significantly higher rates of hypertension, diabetes, and hyperlipidemia compared to the single-vessel disease cohort examined by Qian et al. Moreover, variations in baseline characteristics, including age and NT-proBNP levels, may complicate the evaluation of myocardial microcirculation dysfunction, potentially affecting the determination of the optimal AMR threshold. Additionally, while Qian et al. primarily used univariate analysis and Cox regression, this study employed multivariable logistic regression and Kaplan–Meier survival analysis, thereby providing stronger evidence for AMR as an independent predictor. Furthermore, a retrospective study conducted by Ma et al. showed that the innovative angiography-based AMR technique is an effective method for assessing coronary microvascular dysfunction in patients with hypertrophic cardiomyopathy. The study found that high microvascular resistance, as determined by three-vessel AMR (≥7.04), was linked to a poorer prognosis (23).

AMR reflects the multifaceted impact of microcirculatory dysfunction during myocardial reperfusion, encompassing both structural and functional pathological changes in the microvasculature (7). Microvascular dysfunction significantly contributes to inadequate myocardial reperfusion in patients with STEMI. Elevated AMR is frequently associated with pathological alterations in microvascular structure, including endothelial cell damage, chronic inflammatory responses, increased vascular permeability, and microthrombus formation (11, 24). These alterations exacerbate ischemia-reperfusion injury and elevate the risk of MACE. Furthermore, microcirculatory dysfunction involves structural abnormalities, impaired microvascular regulatory capacity, and hemodynamic instability (25). Under high-resistance conditions, such dysfunction exacerbates the imbalance between myocardial oxygen supply and metabolic demand, thereby intensifying myocardial injury, leading to ischemia and necrosis, and consequently increasing the risk of MACE. Additionally, elevated afterload may contribute to ventricular remodeling, myocardial fibrosis, and other pathological changes associated with an elevated risk of heart failure, repeated heart attacks, and death due to cardiac causes (26).

In this study, multivariate logistic regression analysis demonstrated that diabetes, hyperlipidemia, NT-proBNP levels, reperfusion time, CK/CKMB levels, platelet count, and smoking were significantly associated with AMR. Collectively, these risk factors may compromise microvascular function, leading to direct endothelial damage, structural changes in the microvasculature, and disruptions in microcirculatory regulatory mechanisms (2, 7, 11). Persistent hyperglycemia in diabetes can directly impair microvascular endothelial cells by accelerating the formation of advanced glycation end products. This endothelial dysfunction leads to reduced vascular dilation capacity, increased microvascular wall permeability, and enhanced inflammation and thrombosis, ultimately elevating microcirculatory resistance (27). Furthermore, hyperglycemia triggers the thickening of the microvascular basement membrane, vascular wall sclerosis, and diminished elasticity (28), all of which contribute to impaired coronary microcirculatory function. Hyperlipidemia facilitates the development of atherosclerotic plaques, resulting in microvascular endothelial damage (10). Dysregulated lipid metabolism triggers inflammation, exacerbates microvascular constriction, and diminishes perfusion. Elevated levels of NT-proBNP, an indicator of ventricular pressure overload, frequently signify compromised cardiac function. Myocardial stress provokes the release of cytokines and inflammatory mediators, which can directly impair the coronary microvascular structure and exacerbate endothelial dysfunction. Prolonged reperfusion time is strongly linked to myocardial ischemia-reperfusion injury. Furthermore, the excessive production of reactive oxygen species during reperfusion can impair microvascular endothelial cells, compromise the endothelial barrier, and induce microvascular constriction, leading to increased microcirculatory resistance and unstable blood flow (29). Elevated creatine kinase (CK) and CK-MB levels are indicative of myocardial cell injury, and the subsequent release of pro-inflammatory mediators can exacerbate microvascular endothelial damage through the activation of local inflammation (7, 24, 25), potentially raising AMR levels. A higher platelet count is closely associated with increased blood viscosity and enhanced platelet aggregation. This hypercoagulable state may promote the formation of microthrombi and impair microcirculatory function (30). Smoking independently contributes to CMD and increases microcirculatory resistance through oxidative stress, endothelial injury, and vasoconstriction. The ROS generated by smoking can directly damage endothelial cells, leading to localized inflammation and microvascular constriction. In patients with a postoperative QFR of <0.8, coronary blood flow regulation may be compromised, potentially due to hemodynamic instability and elevated microvascular resistance. A low QFR not only indicates incomplete coronary blood flow recovery but also suggests potential long-term microcirculatory dysfunction.

As a noninvasive, convenient, and highly predictive marker for microcirculatory assessment, AMR holds substantial clinical value not only in the acute management of patients with STEMI but also in guiding MACE prevention strategies following myocardial infarction. Further large-scale studies and clinical validation are necessary to establish AMR as a reliable MACE assessment tool, which could provide robust support for individualized cardiovascular disease management.

### Limitations

This study has several limitations that warrant careful interpretation of the findings. First, its single-center, retrospective design and relatively small sample size may introduce selection bias and unadjusted confounding, thereby limiting the generalizability and robustness of the results. Although multivariate analysis was performed, residual confounding cannot be entirely excluded. Second, the short follow-up duration may have restricted the assessment of AMR's predictive value for long-term MACE. Third, while AMR—derived noninvasively from coronary angiography—offers practical advantages such as avoiding adenosine and pressure wires, its accuracy depends on estimated parameters like flow velocity and vessel length, which may lead to discrepancies when compared to invasive measures such as the IMR. Furthermore, the lack of external validation against gold-standard methods, such as CMR imaging, limits the confidence in its physiological accuracy.

## Conclusion

AMR exhibited independent prognostic value for predicting MACE, with an optimal cutoff of 246.5 mmHg·s/m, enabling early risk stratification by identifying high-risk patients. Moreover, diabetes, hyperlipidemia, and elevated NT-proBNP levels were significantly associated with an increased risk of MACE. Furthermore, a low postoperative QFR was correlated with a higher MACE risk, underscoring the importance of coronary blood flow restoration in improving patient outcomes.

## Data Availability

The raw data supporting the conclusions of this article will be made available by the authors, without undue reservation.
