# Supplementary material for: The independent value and clinical significance of angio-based microvascular resistance in predicting adverse cardiovascular events in patients with acute ST-elevation myocardial infarction
Source: Front Cardiovasc Med. 2025 Sep 25;12:1637251. doi: 10.3389/fcvm.2025.1637251 (PMC12507889; doi:10.3389/fcvm.2025.1637251)
Supplement: Supplementary file 1 [file Datasheet1.docx]

**Calculation of AMR**


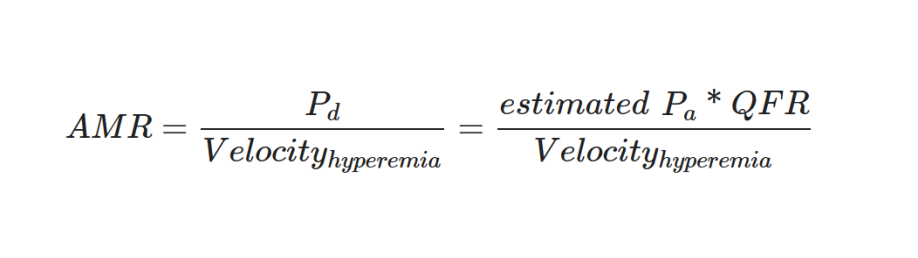


**Schematic Diagram of angio-based microvascular resistance**


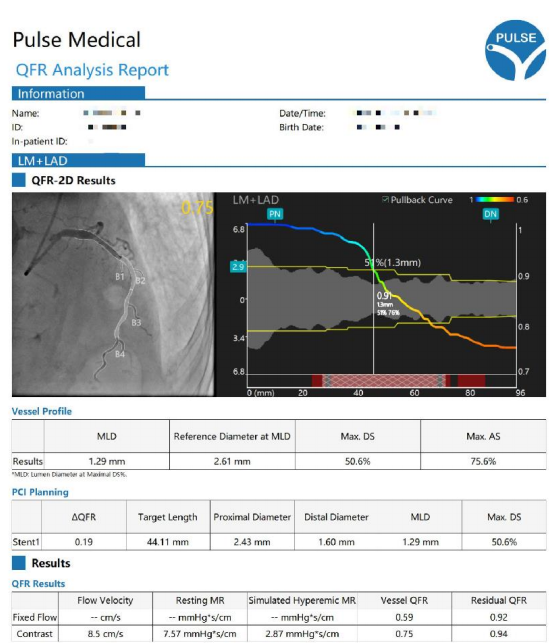


In a 76-year-old female patient, QFR and AMR were quantitatively evaluated immediately after the successful recanalization of the culprit vessel (LAD) via PCI and the achievement of hemodynamic stability. The QFR value was measured to be 0.75, while the AMR was recorded at 287 mmHg·s/m.


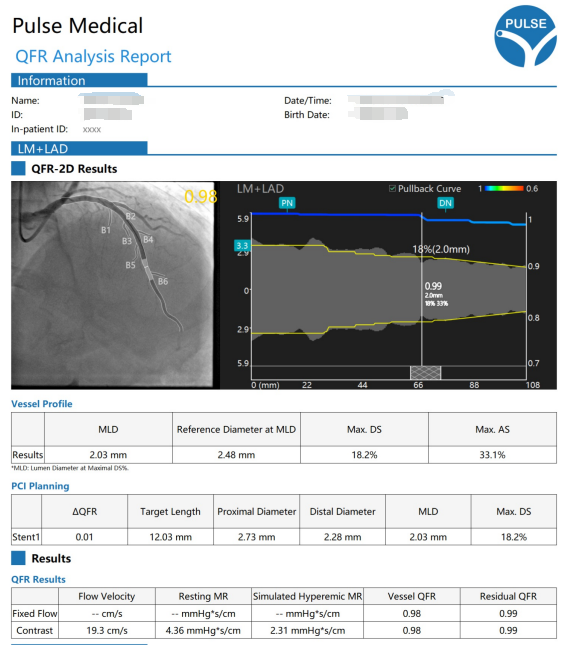


In a 69-year-old female patient, QFR and AMR were quantitatively evaluated immediately after the successful recanalization of the culprit vessel (LAD) via PCI and the achievement of hemodynamic stability. The QFR value was measured to be 0.98, while the AMR was recorded at 231 mmHg·s/m.
